# Ginsenoside Rg5 Ameliorates Cisplatin-Induced Nephrotoxicity in Mice through Inhibition of Inflammation, Oxidative Stress, and Apoptosis

**DOI:** 10.3390/nu8090566

**Published:** 2016-09-13

**Authors:** Wei Li, Meng-Han Yan, Ying Liu, Zhi Liu, Zi Wang, Chen Chen, Jing Zhang, Yin-Shi Sun

**Affiliations:** 1 College of Chinese Medicinal Materials, Jilin Agricultural University, Changchun 130118, China; liwei7727@126.com (W.L.); ymhan@jlau.edu.cn (M.-H.Y.); y.liu@jlau.edu.cn (Y.L.); wangzi8020@126.com (Z.W.); 2 Institute of Agricultural Modernization, Jilin Agricultural University, Changchun 130118, China; lzhiiu@126.com; 3 School of Biomedical Sciences, University of Queensland, Brisbane, Queensland 4072, Australia; Chen.chen@uq.edu.au; 4 Institute of Special Wild Economic Animals and Plant, CAAS, Changchun 132109, China

**Keywords:** ginsenoside Rg5, cisplatin-induced nephrotoxicity, anti-oxidation, anti-inflammation, anti-apoptosis

## Abstract

Although cisplatin is an effective anti-cancer agent that is widely used for treating various types of malignant solid tumors, the nephrotoxicity induced by cisplatin severely limits its clinical application. The present study was designed to explore the potential protective effect of ginsenoside Rg5, a rare ginsenoside generated during steaming ginseng, on cisplatin-induced nephrotoxicity in a mouse experimental model. The possible mechanisms underlying this nephroprotective effect were also investigated for the first time. Rg5 was given at doses of 10 and 20 mg/kg for 10 consecutive days. On Day 7, a single nephrotoxic dose of cisplatin (25 mg/kg) was injected to mice. Cisplatin administration resulted in renal dysfunction as evidenced by increase in serum creatinine (CRE) and blood urea nitrogen (BUN) levels. In addition, cisplatin increased the level of malondialdehyde (MDA) and 4-hydroxynonenal (4-HNE), the makers of lipid peroxidation, and depleted glutathione (GSH) content and superoxide dismutase (SOD) activity in renal tissues. These effects were associated with the significantly increased levels of cytochrome P450 E1 (CYP2E1), 4-hydroxynonenal (4-HNE), tumor necrosis factor (TNF)-α, interleukin (IL)-1β, nuclear factor-kappa B (NF-κB) p65, and cyclooxygenase-2 (COX-2) in renal tissues. However, pretreatment with ginsenoside Rg5 significantly attenuated the renal dysfunction, oxidative stress and inflammation response induced by cisplatin. Furthermore, ginsenoside Rg5 supplementation inhibited activation of apoptotic pathways through increasing Bcl-2 and decreasing Bax expression levels. Histopathological examination further confirmed the nephroprotective effect of Rg5. Collectively, these results clearly suggest that Rg5-mediated alleviation of cisplatin-induced nephrotoxicity may be related to its anti-oxidant, anti-apoptotic and anti-inflammatory effects.

## 1. Introduction

Cisplatin, an important platinum (Pt) containing chemotherapeutic drug, has been widely used to treat various types of solid organ tumors [1]. However, due to its accumulation in renal epithelial cells, nephrotoxicity has been found to be the major dose-limiting side effect of cisplatin [2]. Although the exact mechanism responsible for cisplatin-induced nephrotoxicity is not fully understood, there is growing evidence that multiple cellular and molecular mechanisms were found to be involved [3]. The generation of reactive oxygen species (ROS) and decrease of antioxidant enzymes was found to be involved [4,5]. Excessive ROS generation caused by cisplatin may lead to lipid peroxidation (LPO) and delayed-onset kidney injury. In addition, there is strong evidence that multiple inflammatory mediators are involved in the pathogenesis of cisplatin induced renal damage [6,7]. These mediators include tumor necrosis factor (TNF)-α, interleukin (IL)-1β, nuclear factor-kappa B (NF-κB), cyclooxygenase-2 (COX-2), and inducible nitric oxide synthase (iNOS). Taken together, currently available data indicate that inflammatory response, oxidative stress and pro-apoptotic effect collectively play vital roles in the pathogenesis of cisplatin-induced nephrotoxicity [8].

**Figure 1:**
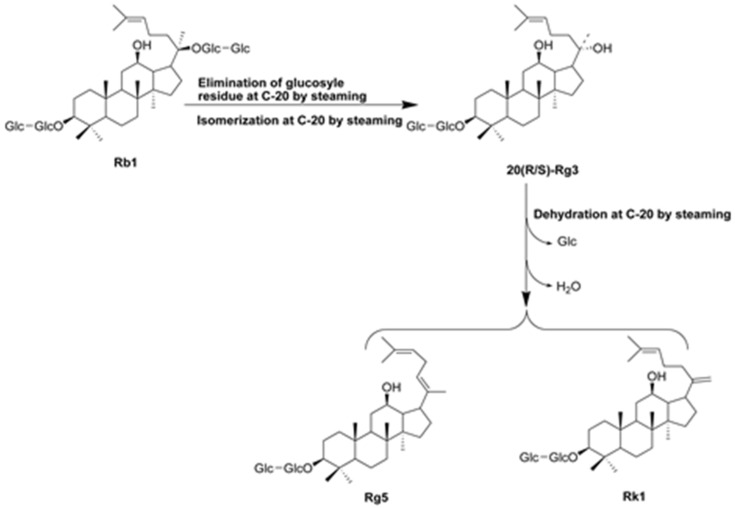
The chemical structure and possible transformation pathway of ginsenoside Rg5. Taking ginsenoside Rb1 as example to show the generation pathway of ginsenoside Rg5.

The use of natural medicines in the prevention and treatment of acute kidney injury has gained considerable popularity. Among these medicines, ginseng (the roots of *Panax ginseng* Meyer), one of the famous Chinese herbal medicines widely used in China, is particularly well-studied [9,10,11,12,13]. Ginsenosides, the major active components in ginseng, possess many pharmacological activities including anti-diabetes, anti-cancer, and anti-inflammation [14,15]. Accumulating evidence indicated that the pharmacological and biological activities of steam-activated ginseng (Red ginseng and Black ginseng) are greater than those of non-steamed ginseng (White ginseng) [16]. Steaming process could cause extensive conversion of ginsenosides in non-steamed ginseng to new less polar degradation ones (Rg3, Rg5, Rk1, Rz1, F4, Rg6, etc.) [17,18]. Ginsenoside Rg5 (Figure 1), a major rare saponin generated during steaming treatment of ginseng, has been shown to exert many pharmacological activities, including anti-inflammation [19,20], anti-cancer [21], anti-dermatitis [22], and memory enhancement [23]. A more recent study suggested that ginseng with microwave-assisted processing (mainly including Rg3, Rg5, and Rk1) exhibited protective effect on cisplatin-induced cytotoxicity in LLC-PK1 renal epithelial cells by regulating inflammation and apoptosis [24]. In addition, Rg5+Rk1 complex were found to significantly ameliorate cisplatin-induced reduction in cell viability. However, there have been no studies to evaluate the protective effect of individual ginsenoside (Rg5 or Rk1) against cisplatin-induced nephrotoxicity in mouse experimental model. Although they are one pair of isomeric ginsenosides, more ginsenoside Rg5 was generated from steamed ginseng than Rk1 using chemical analysis [17]. In addition, ginsenoside Rg5 was easily isolated and purified than Rk1 due to its high contents.

On the basis of the above facts, and taking into account that cisplatin is administered in nephrotoxic doses for more than one cycle to achieve efficacy against solid tumors, we decided to explore the potential ameliorative effect of ginsenoside Rg5 in a mouse model of cisplatin-induced nephrotoxicity. Importantly, to the best of our knowledge, the potential mechanisms underlying such nephroprotective effect of Rg5 were uncovered for the first time.

## 2. Materials and Methods

### 2.1. Reagents and Kits

Cisplatin (purity > 99%) was purchased from Sigma Chemicals (St. Louis, MO, USA). Commercial assay kits for blood urea nitrogen (BUN), creatinine (CRE), glutathione (GSH), superoxide dismutase (SOD), and malondialdehyde (MDA) were purchased from Nanjing Jiancheng Bioengineering Research Institute (Nanjing, China). Two-site sandwich enzyme-linked immunosorbent assays (ELISA) for mouse tumor necrosis factor-α (TNF-α) and interleukin (IL)-1β were obtained from R&D systems (Minneapolis, MN, USA). Hematoxylin and Eosin (H&E) dye kits were acquired from Nanjing Jiancheng Bioengineering Research Institute (Nanjing, China). Hoechst 33258 dye kit was obtained from Shanghai Beyotime Co., Ltd. (Shanghai, China). Rabbit monoclonal anti-Bax, anti-Bcl-2, anti-NF-κB p65, anti-COX-2, anti-4-hydroxynonenal (4-HNE) and anti-cytochrome P450 E1 (CPY2E1) antibodies were provided by Cell Signaling Technology (Danvers, MA, USA). DyLight 488-labeled secondary antibody was provided by BOSTER Bio-Engineer Ltd., Co. (Wuhan, China). Other chemicals were all of analytical grade from Beijing Chemical Factory. TUNEL apoptosis detection kit was provided with Roche Applied Science in Shanghai, China (No. 11684817910).

### 2.2. Sample Preparation

#### 2.2.1. Separation and Purification of Ginsenoside Rg5

Ginseng (*Panax* *ginseng* Meyer) stems-leaves saponins (GSLS) was used for preparing ginsenoside Rg5. Briefly, 10.0 g of GSLS were dissolved fully in the flask containing 1500 mL of 0.1% formic acid solution, and steamed at 120 °C in an autoclave (BXM-30R, Shanghai BOXUN, Shanghai, China) for 2 h. The reaction mixtures were neutralized with saturated NaHCO_3_, extracted with water-saturated n-BuOH, and evaporated in vacuo at 45 °C. The residues were subjected to repeated silica gel column, and eluted with CHCl_3_-MeOH (8.5:1.5) to obtain a mixture of ginsenosides Rk1 and Rg5. Finally, the mixture of Rk1 and Rg5 was separated by semi-preparative High Performance Liquid Chromatography (HPLC) (MeOH:H_2_O = 65:35, 2.5 mL/min) to yield Rg5 with purity of 98.0% (HPLC). The chemical structure of ginsenoside Rg5 was identified through comparison of spectral data of Infrared Ray (IR), Ultra Violet (UV), Nuclear Magnetic Resonance Spectroscopy (NMR), and electrospray ionization mass spectrometry (ESI-MS) with literature values [25].

#### 2.2.2. HPLC Analysis of Ginsenoside Rg5

HPLC were performed with a SHIMADZU LC-20AT system (Tokyo, Japan) , equipped with UV detector. The chromatography was achieved using a Hypersil ODS2 column (4.6 × 250 mm, 5 μm). The column temperature was set at 25 °C and detection wavelength was set at 203 nm. The mobile phase consisted of a mixture of acetonitrile (A) and water (B) with flow rate of 1.0 mL/min. The gradient elution was programmed as follows: 0–30 min, 30%–40% A; 30–37 min, 40%–50% A; 37–45 min, 50%–51% A; 45–60 min, 51%–55% A; 60–75 min, 55%–90% A. The 20 μL of sample solution was directly injected into the chromatographic column manually. The chromatographic peak of ginsenoside Rg5 were confirmed by comparing its retention time with the reference standard.

### 2.3. Animals and Experimental Protocol

Male ICR mice (6 to 8 weeks old), weighing 25–27 g, were provided by Experimental Animal Holding of Jilin University with Certificate of Quality No. of SCXK (JI) 2011-0004 (Changchun, China). Mice were supplied rodent laboratory chow and tap water ad libitum, and maintained under controlled conditions at constant temperature (25 ± 2 °C) and humidity (60% ± 10%) with a 12 h light/dark cycle. All animals handing procedures were performed in strict accordance with the Guide for the Care and Use of Laboratory Animals (Ministry of Science and Technology of China, 2006). All experimental procedures were approved by the Ethical Committee for Laboratory Animals of Jilin Agricultural University (Permit Number: 15-011).

After acclimation for one week, mice were randomly assigned into 4 experimental groups with 8 mice in each group: normal control, cisplatin control, and cisplatin + Rg5 groups (10 and 20 mg/kg, respectively). Ginsenoside Rg5 was dissolved in 0.05% carboxymethylcellulose (CMC) and administered intragastrically at the dose of 10 and 20 mg/kg for 10 days. This dosage was chosen based on our preliminary experiments and other previous studies [19,26]. On the 7th day, animals in cisplatin control and Rg5-treated groups received a single intraperitoneal injection of cisplatin (25 mg/kg) to induce nephrotoxicity in mice.

Mice were anaesthetized with pentobarbital, subsequently sacrificed at 72 h after cisplatin injection (Day 10). Blood samples were collected and then centrifuged at 3000 rpm to separate the serum and stored at −20 °C for determining BUN and CRE levels. Meanwhile, the kidneys were harvested and weighed, the kidney index was calculated by the following formula: kidney index (mg/g) = kidney weight/ body weight. One of the kidneys was immediately kept in 10% neutral buffered formalin and embedded in paraffin for histopathological staining and immunohistochemistry (IHC) analysis. For biochemical estimation, other kidney was snap frozen in liquid nitrogen and stored at −80 °C till analysis.

### 2.4. Assessment of Biochemical Parameters

Two key markers of renal function, blood urea (BUN) and creatinine (CRE), were measured by using commercially available kits according to the manufacturer’s instructions (Nanjing Jiancheng Bioengineering Research Institute, Nanjing, China). 

To assess the oxidative stress markers in kidney tissues, the levels of glutathione (GSH) and superoxide dismutase (SOD) were tested by using commercially available kits according to the manufacturer’s instructions (Nanjing Jiancheng Bioengineering Research Institute, Nanjing, China). Kidney tissues were removed from −80 °C and homogenized in ice-cold 0.1 M phosphate buffer (pH 7.4), the homogenates were filtered and centrifuged using a refrigerated centrifuge at 4 °C. The supernatants obtained were then used to determine enzyme activity [27]. One part of the homogenate was used to assess lipid peroxidation by measuring thiobarbituric acid reactive substances (TBARS) and is expressed in terms of malondialdehyde (MDA) content, a lipid peroxidation marker. The enzyme activity was expressed as unit of activity, or micromole per milligram of protein.

### 2.5. Measurement of Kidney TNF-α and IL-1β Levels

At the end of experiment, kidney tissues were removed and homogenized in ice-cold PBS. Then the homogenates were centrifuged at 3000× *g* for 10 min. The supernatants were kept at −80 °C till measurement. The levels of TNF-α and IL-1β in kidney tissues were measured using commercial ELISA kits (R&D, Minneapolis, MN, USA) according to the manufacturer’s protocols. The absorbance was measured at 450 nm in an ELISA reader (Bio-Rad, Pleasanton, CA, USA). All assays were performed in duplicate.

### 2.6. Histopathological Analysis

For the histopathological analysis, the kidney were instantly dissected out and fixed in 10% neutral buffered formalin solution. After routine processing, the kidney tissues were embedded in paraffin, and cut at a thickness of 5 µm. Sections were stained with hematoxylin and eosin (H&E), and subsequently examined using a light microscope for histopathological examination.

The kidney sections were coded and examined by two independent trained observers, and a minimum of 20 consecutive fields at a magnification of 400× were assessed and scored in each section. Histological changes due to acute tubular necrosis were evaluated in the outer stripe of the outer medulla on H&E-stained tissues as previously described [28,29]. Tubular injure scores were semi-quantitatively analyzed by counting the percent of tubules that displayed cell necrosis, tubule dilatation, loss of brush border, and cast formation as follows: 0, none; 1, <10%; 2, 10% to 25%; 3, 25% to 75%; 4, >75%.

### 2.7. Hoechst 33258 Staining

Hoechst 33258 staining was performed as previously described with some modifications [30]. Briefly, at the end of the experiments, the mice were euthanized and the renal tissues were dissected out and fixed in 10% neutral buffered formalin solution. We randomly chose three samples from each group. Then, these samples were cut into 5 μm sections and stained by Hoechst 33258 (10 μg/mL). After being washed with PBS three times, stained nuclei were visualized under UV excitation and photographed under a fluorescent microscope (Olympus BX-60, Tokyo, Japan).

### 2.8. Immunohistochemistry (IHC) and Immunofluorescence

Immunohistochemical analysis was performed as previously described [31,32]. Briefly, the 5 μm thick paraffin sections were deparaffinized and rehydrated with a series of xylene and aqueous alcohol solutions, respectively. After antigen retrieval in citrate buffer solution (0.01 M, pH 6.0) for 20 min, the slides were washed with Tris-buffered saline (TBS 0.01 M, pH 7.4) for three times and incubated with 1% bovine serum albumin for 1 h. The blocking serum was tapped off, and the sections were incubated in a humidified chamber at 4 °C overnight with primary antibodies against NF-κB p65 (1:200), COX-2 (1:200), Bax (1:400), and Bcl-2 (1:200), followed by secondary antibody for 30 min. Substrate was added to the sections for 30 min followed by dispute adjudication board (DAB) staining and hematoxylin counter-staining. The positive staining was determined mainly by a brownish-yellow color in the nucleus of the cells. The immunostaining intensity was analyzed by light microscopy (Olympus BX-60, Tokyo, Japan). 

To assess the expression of CYP2E1 and 4-hydroxynonenal (4-HNE), a marker of lipid peroxidation, in cisplatin-induced nephrotoxicity, immunofluorescence staining was conducted in kidney tissues of the cisplatin-treated group and the normal control group. Immunofluorescence was carried out on tissue sections processed as described for immunohistochemistry. The sections were incubated overnight at 4 °C with the rabbit anti-mouse CYP2E1 antibody (1:200) and 4-HNE antibody (1:100). The next day, the slides were exposed to the DyLight 488-labeled secondary antibody (BOSTER, Wuhan, China). Nuclear staining was performed using 4,6 diamidino-2-phenylindole (DAPI). Immunofluorescence staining was visualized using a Leica microscope (Leica TCS SP8, Solms, Germany).

### 2.9. TUNEL Assay

For TUNEL assay, an in situ apoptosis detection kit (Roche Applied Science, Shanghai, China) was employed to detect apoptotic cells in the kidney tissues. Briefly, the sections were treated with 20 µg/mL of proteinase K in distilled water for 10 min at room temperature. To block endogenous peroxidase, the slides were incubated in methanol containing 3% hydrogen peroxide for 20 min and sections were incubated with equilibration buffer and terminal deoxynucleotidyl transferase. Finally, the sections were incubated with anti-digoxigenin-peroxidase conjugate. Peroxidase activity in each tissue section was shown by the application of diaminobenzidine. Sections were counterstained with hematoxylin.

### 2.10. Western Blotting Analysis

Western blot analysis was performed as described previously [15]. Briefly, the kidney tissues were lysed in Radio Immunoprecipitation Assay (RIPA) buffer. Equal amounts of protein (50 µg/lane) were analyzed by 12% SDS-PAGE and transferred onto PVDF membrane. The membranes were blocked with 5% non-fat milk in Tris-buffered saline (TBS) with 0.1% Tween-20 and then incubated with the primary antibodies against COX-2 (1:100), Bax (1:2000), and NF-κB (1:1000) at 4 °C overnight. The membranes were then incubated for 1 h at room temperature with the secondary antibodies. Signals were detected using Emitter Coupled Logic (ECL) substrate (Pierce Chemical Co., Rockford, IL, USA). The intensity of the bands was assayed by computer Image plus 6.0 software (Media Cybernetics, Rockville, MD, USA). 

### 2.11. Statistical Analysis

Data are expressed as the Means ± standard deviation (S.D.). Statistical analysis was performed using SPSS 17.0 (SPSS, Chicago, IL, USA). The statistical significance of differences between experimental groups were determined by one-way analysis of variance (ANOVA). *p*-values of less than 0.05 were considered to be significant. Statistical graphs were produced through Graghpad Prism 6.0.4. (Graghpad Software, La Jolla, CA, USA).

## 3. Results

### 3.1. Isolation and Identification of Ginsenoside Rg5

**Figure 2:**
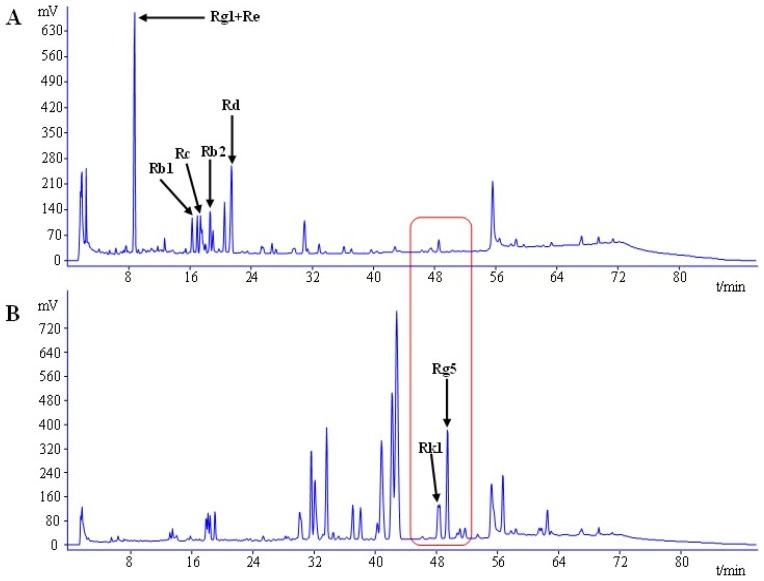
HPLC chromatograms of: GSLS (**A**); and steamed GSLS (**B**).

Since the yield of saponins from stems-leaves was higher than those from the main roots and hair roots, ginseng stems-leaves saponins (GSLS) was used to obtain ginsenoside Rg5 in this study. The changes in ginsenoside compositions and HPLC chromatograms with the steaming of GSLS were shown in Figure 2. The results indicate that ginsenoside compositions changed significantly after steaming treatments. The levels of ginsenosides Rb1, Rb2, Rc, and Rd decreased dramatically under the high temperature. Ginsenoside Rg5, which was not present in GSLS, remarkably increased with the content of 78.56 mg/g after steaming treatment. Interestingly, the findings from our results are in agreement with those reported previously by Kim et al. [33], who employed autoclave steaming of ginseng roots at 120 °C for 2 h to get the highest content of Rg3 and Rg5.

### 3.2. Ginsenoside Rg5 Ameliorates Cisplatin-Induced Renal Dysfunction

As shown in Table 1, single injection of cisplatin with 25 mg/kg resulted in notable weight loss and increased kidney index in the cisplatin-injected group compared with normal group on Day 10 (*p* < 0.05). However, these changes were significantly prevented by ginsenoside Rg5 at dose of 10 and 20 mg/kg (*p* < 0.05 or *p* < 0.01).

To further evaluate whether or not ginsenoside Rg5 preserves renal function, serum creatinine (CRE) and BUN levels were measured in all groups. After cisplatin injection, the levels of CRE and BUN, the hallmarks of kidney damage, were both dramatically elevated (*p* < 0.05 or *p* < 0.01), reflecting a severe damage to kidney. Pretreatment with Rg5 at the doses of 10 and 20 mg/kg exerted a dose-dependent renoprotective effect as demonstrated by normalization of CRE and BUN compared to cisplatin control group.

### 3.3. Ginsenoside Rg5 Attenuates Cisplatin-Induced Oxidative Stress 

**Figure 3:**
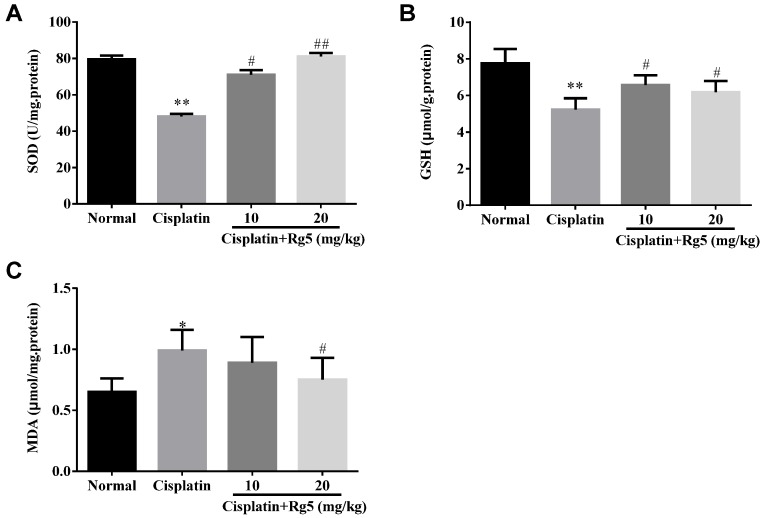
Effects of Rg5 on the levels of: SOD (**A**); GSH (**B**); and MDA (**C**) in cisplatin-induced nephrotoxicity. All data were expressed as mean ± S.D., *n* = 8. * *p* < 0.05, ** *p* < 0.01 vs. normal group; ^#^ *p* < 0.05, ^##^ *p* < 0.01 vs. cisplatin group. GSH = glutathione; SOD = superoxide dismutase; MDA = malondialdehyde.

**Figure 4:**
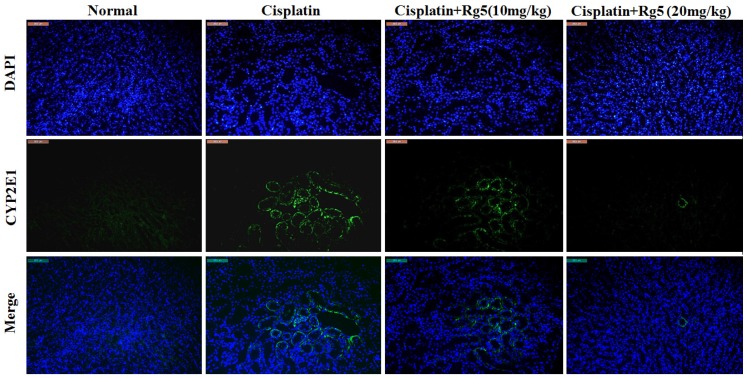
Effects of ginsenoside Rg5 on the expression of cytochrome P450 E1 (CYP2E1). The expression level of CYP2E1 (**green**) in tissue section isolated from different groups was assessed by immunofluorescence. Representative immunofluorescence images were taken at 400×. 4′,6-Diamidino-2-phenylindole (DAPI) (**blue**) was used as a nuclear counterstain.

**Figure 5:**
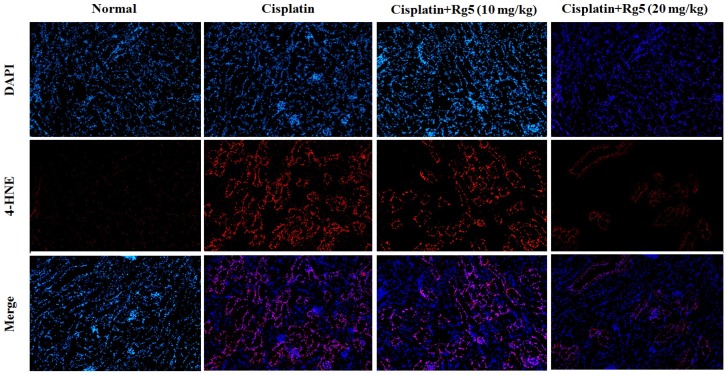
Effects of ginsenoside Rg5 on the expression of 4-hydroxynonenal (4-HNE). The expression level of 4-HNE (**Red**) in macrophages isolated from different groups was assessed by immunofluorescence. Representative immunofluorescence images were taken at 400×. 4’,6-Diamidino-2-phenylindole (DAPI) (**blue**) was used as a nuclear counterstain.

As mentioned earlier, oxidative stress damage is involved in the mechanisms of cisplatin-induced nephrotoxicity [34]. As shown in Figure 3, cisplatin exposure resulted in dramatic decrease of GSH content and SOD activity along with increase of MDA, compared to the normal group (*p* < 0.01). In contrast, pretreatment with 10 and 20 mg/kg of ginsenoside Rg5 for 10 days significantly decreased MDA level and restored the antioxidant status as demonstrated by increase in GSH content and SOD activity (*p* < 0.05). These data suggest that ginsenoside Rg5 alleviated oxidative injury in kidney via up-regulating anti-oxidant enzyme activity. Concomitantly, the expression of the CYP2E1 metabolizing enzyme, which was increased by cisplatin, was reduced by Rg5 in a dose-dependent manner (Figure 4). These results suggest that administration of Rg5 protects the kidneys from cisplatin-induced oxidative stress. To verify whether oxidative stress is related to the development of cisplatin-induced nephrotoxicity in vivo, lipid peroxidation was confirmed using 4-HNE staining. At 72 h after cisplatin treatment, strong 4-HNE fluorescence intensities were detected in the tubular epithelium of the kidney of mice treated with cisplatin alone. However, Rg5 pretreatment for seven days significantly decreased fluorescence intensities, especially in the group with dosage of 20 mg/kg. The sites of lipid peroxidation were highly correlated with the necrotic regions in the kidney. Consistent with the 4-HNE staining, quantitative MDA analysis showed an increase of lipid peroxidation after cisplatin exposure, but this was significantly blocked by pretreatment with Rg5 (Figure 5).

### 3.4. Ginsenoside Rg5 Attenuates Cisplatin-Induced Renal Inflammation

**Figure 6:**
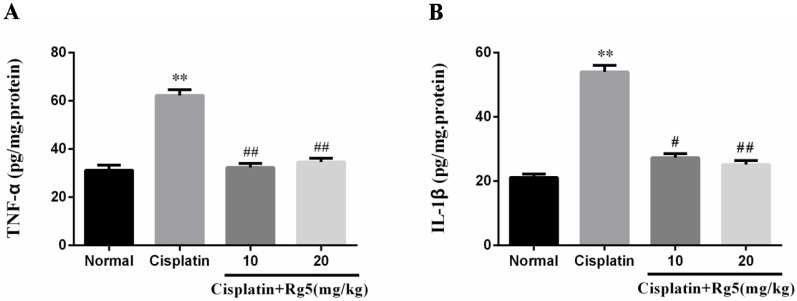
Effect of Rg5 on the levels of renal inflammatory cytokines in cisplatin-induced nephrotoxicity. The levels of: tumor necrosis factor (TNF)-α (**A**); and interleukin (IL)-1β (**B**) in kidney tissues were determined by ELISA kits. All data were expressed as mean ± S.D., *n* = 8. ** *p* < 0.01 vs. normal group; ^#^ *p* < 0.05, ^##^ *p* < 0.01 vs. cisplatin group.

Previous studies indicated that oxidative stress is known to be associated with release of pro-inflammatory cytokine such as TNF-α and IL-1β in cisplatin-induced nephrotoxicity [28]. In the present study, the levels of TNF-α and IL-1β in kidney tissues were determined by ELISA. As indicated in Figure 6, single cisplatin injection (cisplatin control group) caused markedly higher levels of TNF-α and IL-1β in kidney tissues than those in normal group (*p* < 0.05). In contrast, pretreatment with ginsenoside Rg5 significantly suppressed the production of TNF-α and IL-1β in a dose-independent manner (*p* < 0.05).

**Figure 9:**
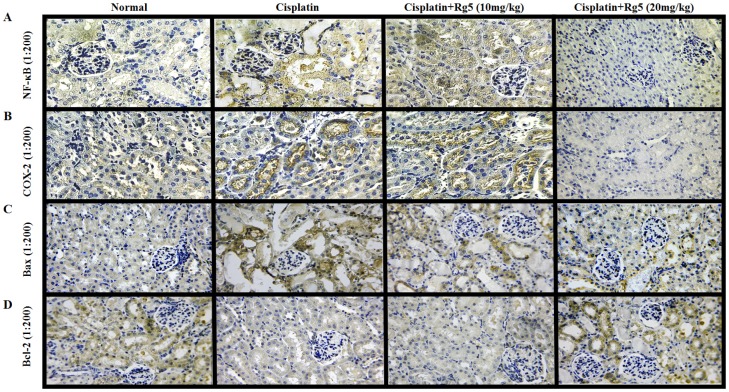
Effects of ginsenoside Rg5 on the expression of: nuclear factor-kappa B (NF-κB) p65 (**A**); cyclooxygenase-2 (COX-2) (**B**); Bax (**C**); and Bcl-2 (**D**) (×200). The protein expression was examined by immunohistochemistry in kidney tissues from normal, cisplatin, cisplatin + Rg5 (10 mg/kg), and cisplatin + Rg5 (20 mg/kg).

**Figure 10:**
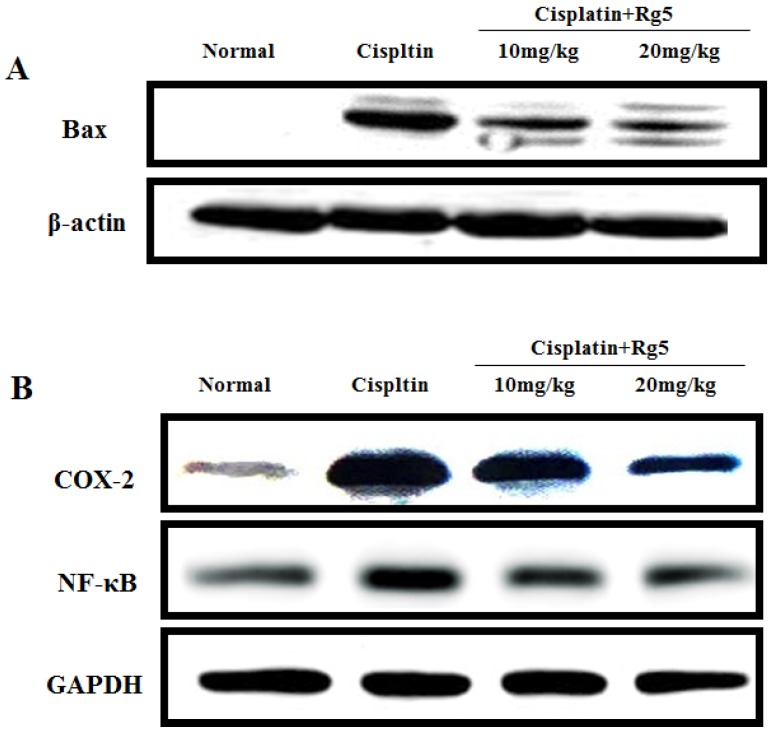
Effects of ginsenoside Rg5 on the protein expression of: Bax (**A**); and nuclear factor-kappa B (NF-κB) p65, and cyclooxygenase-2 (COX-2) (**B**). The protein expression was examined by Western blotting analysis in kidney tissues from normal, cisplatin, cisplatin + Rg5 (10 mg/kg), and cisplatin + Rg5 (20 mg/kg).

To gain a better understanding of the anti-inflammatory effects of Rg5, we measured the expression levels of NF-κB p65 and COX-2 in each treatment group using immunohistochemical analysis and Western blotting analysis. As shown in Figure 9 and Figure 10, the expression of NF-κB p65 and COX-2 were negligible in the kidney tissue sections of normal group. However, the positive area of NF-κB p65 and COX-2 expression in the cisplatin control group was significantly increased compared to normal group. Importantly, administration of Rg5 for 10 days resulted in dose-dependent reduction of NF-κB p65 and COX-2 expression.

### 3.5. Ginsenoside Rg5 Attenuates Cisplatin-Induced Renal Histopathological Changes

**Figure 7:**
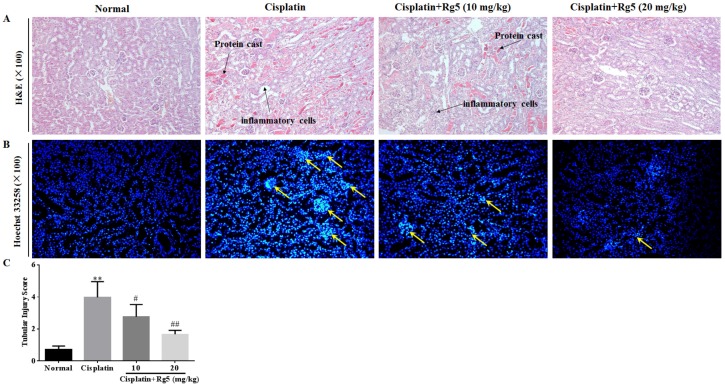
Histological examination of morphological changes in kidney tissues. Renal tissues stained with: hematoxylin-eosin (H&E) (100×) (**A**); and Hoechst 33258 (100×) (**B**); and the tubular injury scores (**C**). Arrows show necrotic and injured epithelial cells, stars show cast formation. All data were expressed as mean ± S.D., *n* = 8. ** *p* < 0.01 vs. normal group; ^#^ *p* < 0.05, ^##^ *p* < 0.01 vs. cisplatin group.

Light microscopy of kidney tissues in normal mice revealed normal morphology of tubules with no evidence of inflammation, cell necrosis, and cast formation (Figure 7A,C). Kidney tissues in cisplatin injected mice showed tubular necrosis, denudation of epithelium, cast formation, and interstitial inflammation. In the group treated with low dose of ginsenoside Rg5 (10 mg/kg), there was remarkable tubular damage and infiltration of inflammatory cells. However, at the higher dose of ginsenoside Rg5 (20 mg/kg), tubules markedly appeared histologically normal and no inflammation and cast formation was observed in kidney tissues. These observations confirm the earlier result that Rg5 exerted the anti-inflammatory action.

In order to determine whether ginsenoside Rg5 treatment decreased renal tubular cell apoptosis in cisplatin-induced acute kidney injury (AKI), Hoechst 33258 staining was used to observe apoptosis of renal tubular cells in the present study. As depicted in Figure 7B, renal tubular cells in cisplatin control group were observed as significant nuclear fragmentation and condensation, indicating apoptosis of renal tubular cells. However, after ginsenoside Rg5 treatment, most cell nucleus exhibited the round-shaped nuclei with homogeneous fluorescence intensity and regular contours.

**Figure 8:**
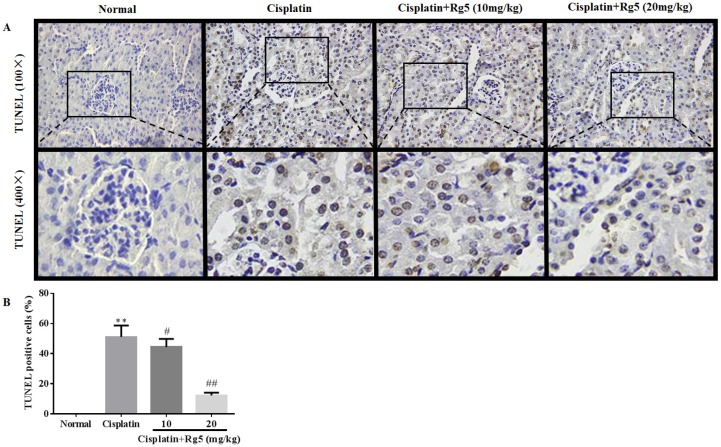
Histological examination of morphological changes in kidney tissues. Renal tissues stained with TUNEL (100× & 400×) (**A**). The presence of TUNEL positive cells were measured by the image analyzer (**B**). All data were expressed as mean ± S.D., *n* = 8. ** *p* < 0.01 vs. normal group; ^#^ *p* < 0.05, ^##^ *p* < 0.01 vs. cisplatin group.

To further determine whether apoptosis would coexist with necrosis in cisplatin-induced nephrotoxicity, the apoptosis in renal tubular cells was confirmed and quantified using TUNEL staining. As depicted in Figure 8, almost no apoptotic cells were observed in renal tissues of normal groups. Compared to normal group, the number of TUNEL positive cells was significantly increased in the cisplatin-induced group. However, Rg5 pretreatment (20 mg/kg) significantly reduced the number of TUNEL positive cells in renal tissues.

### 3.6. Ginsenoside Rg5 Ameliorates Cisplatin-Induced Tubular Apoptosis

In order to further measure the extent of apoptosis in renal tissues, we examined the impacts of ginsenoside Rg5 on the anti-apoptotic factor Bcl-2 and the pro-apoptotic factor Bax in all experimental groups using immunohistochemical analysis. As shown in Figure 9C,D, Bax positive expression was heterogeneous but was located in the cytoplasm of tubular cells. The rate of positive expressions of Bax were found to be significantly lower in Rg5 pretreatment groups than in cisplatin control group. Bcl-2 positive expression was also observed in the cytoplasm of tubular cells, and the expression of Bcl-2 has a significant increase in cisplatin + Rg5 (20 mg/kg) groups compared to cisplatin control group.

In addition, Western blotting was used to analyze the pro-apoptotic factor Bax. As depicted in Figure 10B, Rg5 treatment with 10 and 20 mg/kg increased the protein expression of Bax. These results are consistent with that in immunohistochemical analysis.

## 4. Discussion

Cisplatin is one of the most important chemotherapeutic agents used in the treatment of several solid tumors. Although cisplatin provides better clinical response by suppressing cancer growth, higher doses of cisplatin also induced severe nephrotoxicity through the induction of oxidative stress, inflammation, and apoptosis. Unfortunately, to date there is no drugs available that can protect the kidney from this deleterious effect of cisplatin.

The raw and steamed ginseng (*P*. *ginseng* Meyer*)* was known to show distinct chromatographic profiles, indicating that steaming process altered the composition of ginsenosides by increasing the generation of non-polar ginsenosides [35]. Ginsenosides in the raw *P*. *ginseng* underwent hydrolysis to form the rare ginsenosides during steaming process [33]. For example, ginsenosides Rk1 and Rg5 were produced from protopanaxadiol-type ginsenosides including Rb1, Rb2, Rc, Rd, and others through deglycosylation at C-20 was partially detached [36]. In recent years, it was found that steamed ginseng (e.g., red ginseng and black ginseng) exhibited more remarkable pharmacological activities and therapeutic efficacy than non-steamed one (e.g., white ginseng) [16]. The low-polar ginsenosides from black ginseng exerted more strong biological activities including anti-oxidative, anti-inflammatory and anti-cancer activities [37]. As we know, ginsenoside Rg5 is one of major rare saponins of steamed ginseng and exerted many pharmacological effects including anticancer, radical scavenging, and neuroprotective activities [19,22,23,38]. Although a recent study reporting the protective effects of ginseng with microwave-assisted processing (mainly contain ginsenoside Rk1 and Rg5) on cisplatin-induced nephrotoxicity in LLC-PK1 cells [24], the efficacy and relative mechanism of Rg5 itself on cisplatin-induced nephrotoxicity in mice has not been reported so far. 

In the present study, we found that ginsenoside Rg5 attenuated cisplatin-induced nephrotoxicity, thereby reducing renal tubular damage, decreasing oxidative stress, and suppressing apoptosis and inflammation. The results show that intraperitoneal administration of 25 mg/kg cisplatin resulted in increased levels of CRE and BUN, suggesting infliction of acute renal damage after cisplatin exposure. Currently, we observed that treatment with ginsenoside Rg5 at the dose of 10 and 20 mg/kg significantly prevented the rise and markedly attenuated cisplatin-induced renal dysfunction which was further confirmed by biochemical assay and tissue morphological examination.

Numerous studies in animal models have demonstrated that oxidative stress acts as an important pathogenic factor in causing cisplatin-induced AKI [39]. It is well known that cisplatin administrations are associated with increased formation of free radicals, and with heavy oxidative stress and lipid peroxidation. In the present investigation, the kidney SOD activity was significantly decreased in the cisplatin-treated mice. In addition, the level of MDA, an important marker of lipid peroxidation, increased in the cisplatin-intoxicated mice kidney. Ginsenoside Rg5 treatment decreased lipid peroxidation, and restored antioxidant capacity of kidney by elevating SOD activity and GSH content. Our observations also gain support by a study conducted by Bao et al., in which they have shown that ginsenoside Rg5 attenuated ethanol induced oxidative stress damage in primary cultured rat cortical cells through its anti-oxidant effect [40]. Prior to our work, a report by Liu et al. clearly showed that drug-metabolizing enzyme CYP2E1 plays an important role in CP-induced cytotoxicity by severing as a site for the generation of reactive oxygen species (ROS) including hydrogen peroxide and hydroxyl radical [41]. Interestingly, overexpression of CYP2E1 in renal tissues of cisplatin-treated mice, in the present investigation, agrees with the previous findings. However, ginsenoside Rg5 reversed the cisplatin-mediated increase in CYP2E1 expression. Consistent with the 4-HNE staining, quantitative MDA analysis also showed an increase of lipid peroxidation after cisplatin exposure, but this was significantly blocked by pretreatment with Rg5.

In addition to oxidative stress, recent evidence indicated that inflammation plays an important role in the pathogenesis of cisplatin-induced renal injury [41]. NF-κB is a ubiquitous protein complex participating in the regulation of cell signals in the body under various conditions. Previous studies showed that cisplatin activates the NF-κB pathway, promoting the transcription of genes encoding a series of inflammatory cytokines including TNF-α, IL-1β, and COX-2 [42,43]. Rg5 was also reported to inhibit the mRNA expression of COX-2 via suppression of the DNA binding activities of NF-κB p65 in lipopolysaccharides (LPS)-stimulated BV2 microglial cells [38]. These results are consistent with our study, cisplatin alone group mice showed markedly increased in expression of NF-κB p65 and COX-2. In contrast, Rg5 pretreated group mice showed declined expression of NF-κB p65 and COX-2. TNF-α, a prototypical inflammatory cytokine that heralds apoptosis of epithelial cell, is thought to play a central role in mediating renal injury [44]. TNF-α lines the tubular structure through death receptor pathway, and induces apoptosis and ROS production [45]. The induction of cyclooxygenase (COX)-2, an inducible form of COX, can occur during tissue damage or inflammation in response to cytokines. It has been proved that COX-2 plays a pivotal role in cisplatin-induced acute mouse renal lesions [46]. Ginsenoside Rg5 was shown in the current study to markedly suppress the cisplatin-induced elevation in inflammation cytokines including TNF-α and IL-1β, clearly indicating that Rg5 exerted an anti-inflammatory effect in cisplatin-induced renal injury. Interestingly, the results of the current study are consistent with a previous report that Rg5 ameliorated lung inflammation by inhibiting the expression of pro-inflammatory cytokines IL-1β and TNF-α, as well as inflammatory enzymes COX-2 and iNOS in LPS-stimulated alveolar macrophages [19].

Accumulating evidence indicated that cisplatin-induced renal injury is associated with apoptosis. Specifically, apoptosis is an important mode of cell death in cisplatin nephrotoxicity, and numerous studies have demonstrated renal tubular cell apoptosis after cisplatin treatment [42]. There were two of the most important members concerning apoptosis in Bcl-2 family including the pro-apoptotic protein Bax and the anti-apoptotic protein Bcl-2 [47]. The binding of extracellular TNF-α to a cell surface receptor up-regulates the pro-apoptotic protein Bax and down-regulates the anti-apoptotic protein Bcl-2 [48]. In the present study, the findings from immunohistochemistry analyses of kidney tissues clearly revealed that the expression of Bax was significantly inhibited while the expression of Bcl-2 was relatively increased, indicating that ginsenoside Rg5 exerts anti-apoptotic properties in the context of cisplatin nephrotoxicity. These data indicate that Rg5 could dramatically inhibit apoptosis of tubular cells in cisplatin-induced nephrotoxicity. Importantly, the anti-apoptotic effect of ginsenoside Rg5 + Rk1 complex has been reported by Park et al., using LLC-PK1 renal epithelial cells as the in vitro model [24].

Due to the hemolytic action of ginsenosides, they are typically administered orally. After being taken, these ginsenosides are exposed to gastric juices, digestive enzymes, and bacterial enzymes, only a small group of ginsenosides are absorbed from the intestines after biotransformation by human intestinal bacteria. It is reported that the absorption rate of ginsenoside is as low as 0.1% to 3.7% [49]. Recently, Ryu et al., reported that ginsenoside metabolites including Rg3, Rg5, and Rk1 showed greater bioavailability than original ginsenoside such as Rb1, Rb2, and Rd [50]. In the present work, the result that ginsenoside Rg5 with low dosage of 10 mg/kg daily can exhibit so good protective effect on cisplatin-induced nephrotoxicity may be due to its good absorption. Recently, Liang et al., have reported that ginsenoside Rg5 had marked genotoxic effects on five human cervical cancer cell lines [51], however, it exerted contrast cisplatin’s genotoxicity on kidney. This may be because ginsenosides show bidirectional regulation effect on body.

## 5. Conclusions

In conclusion, the findings of the present investigation support that ginsenoside Rg5 treatment reduced functional, biochemical and morphological changes in cisplatin-induced nephrotoxicity in mice. Rg5 acts on multiple mechanisms that result in the alleviation of cisplatin-induced nephrotoxicity including attenuating of oxidative stress, suppression of inflammation, and inhibition of apoptosis in cisplatin-treated mice. Since ginsenoside Rg5 can be prepared from protopanaxadiol-type saponins using easy steaming process, it has the potential to be used as a dietary supplement to ameliorate nephrotoxic side effect of cisplatin. Certainly, the favorable profile of ginsenoside Rg5 may be further explored and corroborated in more studies to establish its recommendation for clinical use in patients undergoing treatment with cisplatin.

## Figures and Tables

**Table 1:** Effects of ginsenoside Rg5 on body weight change, kidney index and serum markers in cisplatin-induced acute kidney injury.

Groups	Dosage (mg/kg)	Cisplatin Dosage (mg/kg)	Body Weight Change (g)	Kidney Index (mg/g)	BUN (mmol/L)	CRE (µmol/L)
Normal	—		+6.86	1.54 ± 0.14	7.53 ± 1.32	31.52 ± 2.14
Cisplatin	—	25	−2.80 *	2.35 ± 0.15 *	14.20 ± 2.11 **	201.34 ± 6.23 **
Cisplatin + Rg5	10	25	−1.09	1.91 ± 0.12 ^#^	12.80 ± 1.36 ^#^	97.96 ± 3.12 ^#^
	20	25	+0.64 ^#^	1.56 ± 0.14 ^##^	11.70 ± 1.05 ^##^	45.00 ± 2.15 ^##^

BUN = Blood urea; CRE = creatinine. Values are expressed as the mean ± S.D., *n* = 8. * *p* < 0.05, ** *p* < 0.01 vs. normal group; ^#^ *p* < 0.05, ^##^ *p* < 0.01 vs. cisplatin group.

## Abbreviations

The following abbreviations are used in this manuscript:

AKI	Acute kidney injury
SOD	Superoxide dismutase
BUN	Blood urea nitrogen
MDA	Malondialdehyde
CRE	Creatinine
IL-1β	Interleukin-1β
GSH	Glutathione
TNF-α	Tumor necrosis factor-α
